# Assessment of geospatial and hydrochemical interactions of groundwater quality, southwestern Nigeria

**DOI:** 10.1007/s10661-018-6799-8

**Published:** 2018-06-28

**Authors:** PraiseGod Chidozie Emenike, Chidozie Charles Nnaji, Imokhai Theophilus Tenebe

**Affiliations:** 1 Cranfield Water Science Institute, School of Water, Energy and Environment, Cranfield University, Bedfordshire, MK43 0AL UK; 2 Department of Civil Engineering, Covenant University, Ota, Ogun State Nigeria; 3 Department of Civil Engineering, University of Nigeria, Nsukka, Enugu State Nigeria

**Keywords:** Groundwater, Abeokuta, Hydrochemical, Pollution, Geospatial, Southwestern Nigeria

## Abstract

Groundwater pollution resulting from anthropogenic activities and poor effluent management is on the rise in Nigeria. Hence, groundwater used for domestic purposes is questionable and therefore calls for scientific scrutiny. Investigation of hydrochemical interactions and quality of groundwater resource is essential in order to monitor and identify sources of water pollutants. As a result, groundwater samples were collected from 21 locations in Abeokuta South, Nigeria and analyzed for physicochemical parameters using standard methods. Results obtained were subjected to hydrochemical and geospatial analyses. Water quality parameters investigated exhibited wide variations from location to location. Fe^2+^, Mg^2+^, SO_4_^2−^, Cl^−^, total hardness (TH), Mn, Na^+^, NO_3_^−^, SiO_2_, and alkalinity exhibited the highest levels of variation with coefficients of variation of 131.3, 92.8, 83.9, 76.7, 65.9, 64.3, 57.6, 57.2, 57.0, and 52.5, respectively. The average pH value was 6.76 with 71% of the water samples being slightly acidic. Na^2+^, Mg^2+^, Fe^2+^, and EC contents exhibited the most violation of drinking water standards with percent violations of 100, 52.4, 47.6, and 47.6%, respectively. Parameters, such as Mn, Ca^2+^, NO_3_^−^, and CO_3_^2−^, were within the WHO guideline values for drinking water in all the samples. The highest level of significant correlation was found to exist between Na^+^ and Cl^−^ (*r* = 0.84, *α* = 0.01). Six principal components, which explained 83.5% of the variation in water quality, were extracted with the first (34.1%) and second components (15.7%) representing the influence of mineral dissolution and anthropogenic practices, respectively, on the hydrochemistry of the area. Four hydrochemical clusters were identified with distinctly partitioned water quality. Further analysis revealed that 38, 29, 24, and 9% of the samples were the Na-K-HCO_3_, Na-K-Cl-SO_4_, Ca-Mg-HCO_3_, and Ca-Mg-Cl-SO_4_ types, respectively. Anthropogenic activities are increasing threat to groundwater quality in the study location and therefore call for urgent attention. There is also a need for routine monitoring of groundwater in Abeokuta.

**Electronic supplementary material:**

The online version of this article (10.1007/s10661-018-6799-8) contains supplementary material, which is available to authorized users.

## Introduction

Unsafe drinking water is of growing concern and has been attracting global attention (WHO and UNICEF, 2014). Groundwater, a valuable resource, is faced with contamination and depletion due to the propagation of civilization and development near water resource (Emenike et al. 2017a, b; Gao et al. 2010; Karkra et al. 2016). Recently, a report published by WHO indicated that groundwater contamination is partly responsible for the death of 1.7 million children below the age of 5 years annually (WHO, 2017). This explains the fact that the socioeconomic status and health evaluations of many nations have been linked to the development of quality water resources (Akoto et al. 2017; Karikari and Ansa-Asare 2006). The hydrochemistry of any groundwater is heavily dependent on water recharge, precipitation of minerals, soil interaction, dissolution of basement rocks, complementary action from other aquifers, and anthropogenic sources (Aly 2015; Das and Nag 2017; Pazand et al. 2012; Vázquez-Suñé et al. 2005). Various researchers have employed a wide range of analytical methods in order to understand the variability of water quality in groundwater aquifers (Arulbalaji and Gurugnanam, 2017; Barzegar et al. 2017; Edjah et al. 2017; Fijani et al. 2017; Rao et al. 2015; Chandrasekar et al. 2014; Selvakumar et al. 2014; Maiti et al. 2013; Wang, 2013; Chen et al. 2011; Giridharan et al. 2008). Most of these studies showed interesting results and also differentiated the effect of anthropogenic and natural sources on groundwater quality by interrelationships among ions present in water. Such studies have been of immense use in ascertaining the status of groundwater and their suitability for agricultural and domestic applications, as well as the level of treatment required before use (Assubaie 2015; Cao et al. 2014; Emenike et al. 2016; Golchin and Moghaddam 2016; Nazeer et al. 2014; Rasool et al. 2016; Tenebe et al. 2016; Zaidi et al. 2016).

The integration of geographical information system (GIS) and remote sensing (RS) tools have been adopted by other investigators to assess the resource potentials of groundwater reservoirs (Pinto et al. 2017; Ayele et al. 2014; Salari et al. 2014; Junge et al. 2010). GIS and RS tools have proven to be an effective tool to ascertain the accuracy of water quality and monitoring. Also, the combination of thematic layer maps, GIS, and RS tools has made it easier to understand the groundwater chemistry without depending on the lineament component alone. Furthermore, statistical tools such as hierarchical cluster analysis (HCA), principal component analysis (PCA), and cluster groupings (CG) have been accepted as explanatory techniques for analyzing the sources of groundwater and their pollution route (Selvakumar et al. 2014; Yidana, 2010).

In Abeokuta, Ogun State—Nigeria, groundwater sources are being exploited to meet the daily water demands for domestic and agricultural purposes (Odjegba et al. 2015; Adekunle et al. 2013). This situation is further exacerbated by anthropogenic activities resulting in the pollution of ground and surface water resources. The major industrial activities in Abeokuta that are responsible for water pollution included abattoirs, textile mills, sawmills, food processing industries, automobile workshops, as well as large volumes of solid waste generated daily and indiscriminately disposed of in the municipality. The health condition of the inhabitants is tied to environmental conditions, sanitation, and surrounding circumstances, but to maintain a healthy living, it is vital to ensure that the quality of water consumed complies with stipulated drinking water standards. Hence, consistent and proper monitoring is necessary.

In the area, less attention has been given to the hydrochemical analysis of the concentration of groundwater quality parameters. Several researchers have reported the general water characteristics in Abeokuta (Adekunle et al. 2013; Amori et al. 2013; Gbadebo, 2012; Taiwo, 2012). However, a holistic geochemical study integrating water quality parameters, GIS, and RS has not been reported in the literature. Therefore, it is important to understand the spatial variability of groundwater sources within the region. This study was aimed at assessing groundwater quality and hydrochemical interactions in the district and ascertaining the inter-relationship of water quality parameters for potential pollution source identification, using RS, GIS, and statistical tools.

## Materials and methods

### Study area

The sampling sites for the study were in Abeokuta—the capital of Ogun state in Southwestern, Nigeria. Abeokuta is located near a cluster of rocky outcrops on the east bank of River Ogun. It lies between latitude 7.23° N and longitude 3.42° E. The population of Abeokuta is estimated at 451,607 with an annual growth rate of 3.5% (National Population Commission 2010; Ogbiye et al. 2018). The city is joined to Lagos by railway (77 km) or by water (130 km). Other neighboring towns that share common boundaries with Abeokuta include Ketou, Ilaro, Ibadan, Iseyin, and Shagamu.

### Sample collection

**Fig. 1:**
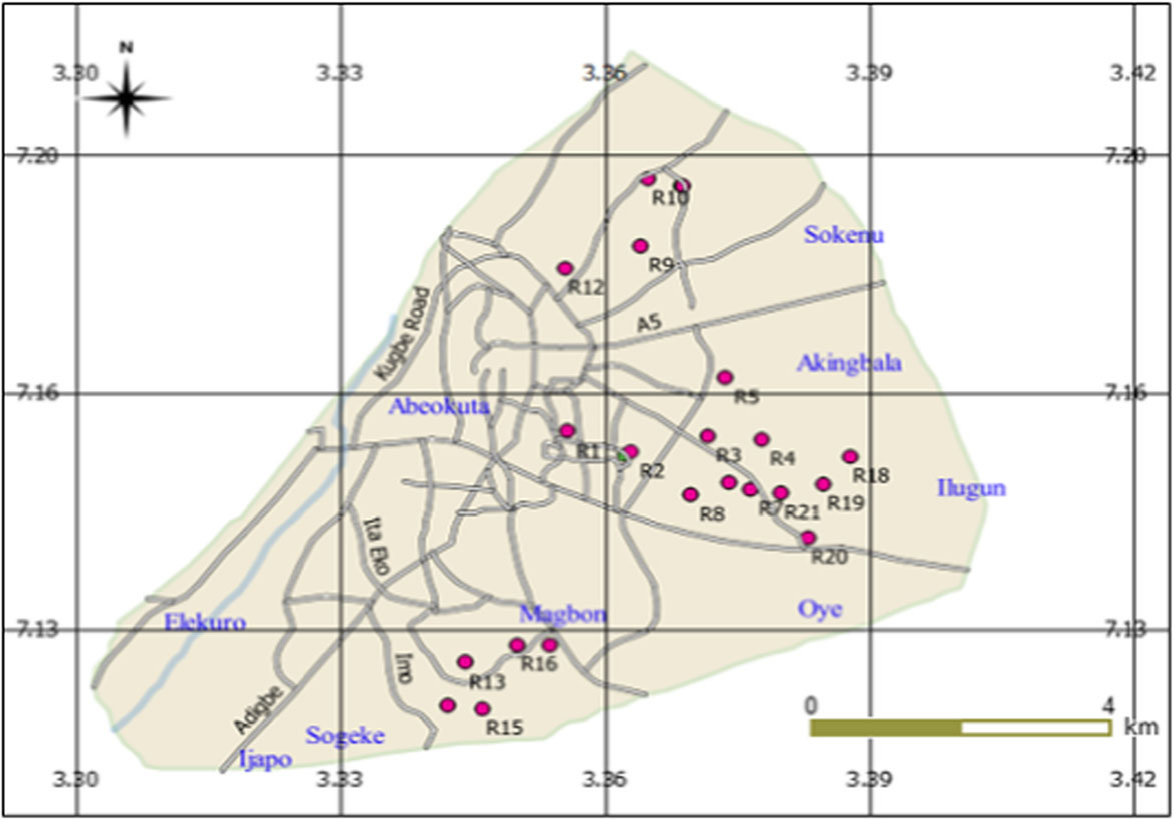
Map of study area showing sampling points

Twenty-one groundwater samples collected from Abeokuta South (Fig. 1) during September 2016 from taps located in 21 different locations (R1–R21) were analyzed in this study. The taps used in this study get their supply from boreholes. Each tap was allowed to run for 10–15 min, and three samples were collected to obtain the mean value of each physicochemical parameter.

Before obtaining the samples, polyethylene containers with screw cocks were rinsed with distilled water having 20% HNO_3_. The polyethylene bottles were further rinsed with distilled water and air-dried before taking it to the collection site. On the collection site, the pre-washed polyethylene containers were rinsed three times with the tap water before representative samples were obtained. The representative samples were labeled correctly and transported to a laboratory in an ice box and later transferred to a refrigerator calibrated to 4 °C.

Field measurements of water quality parameters include hydrogen ion concentration (pH), total dissolved solids (TDS), temperature (temp.), electrical conductivity (EC), and alkalinity (Alka.). The field measurements were performed using a multiparameter Hanna edge HI2030 EC/TDS/salinity meter and Hanna HI98130 probe. Other water parameters, such as dissolved silica (SiO_2_), major anions (carbonates (CO_3_^2−^), bicarbonates (HCO_3_^−^), chloride (Cl^−^), nitrates (NO_3_^−^), and sulfates (SO_4_^2−^)), and major cations (calcium (Ca^2+^), sodium (Na^+^), magnesium (Mg^2+^), and potassium (K^+^)) were measured using standard procedures laid out by the American Public Health Association (APHA, 2005). The concentration of iron (Fe^2+^) and manganese (Mn) was measured under a standard operating condition with a flame atomic absorption spectrophotometer (Perkin Elmer PinAAcle500), and the fluoride ion (F^−^) was measured using a calibrated potentiometric ion-selective electrode (HI5315 reference electrode attached to Hanna HI98191 professional water-resistant portable pH/ISE/ORP meter).

### Data analysis

Laboratory results were subjected to descriptive statistical analyses. The degree of violation of each water quality parameter was estimated by considering the number of times it exceeded the WHO water quality guidelines. Correlation between parameters was also performed using Pearson’s pairwise correlation at 0.05 and 0.01 levels of significance. A geospatial map of each physicochemical parameter was produced using QGIS 2.18.11. PCA was used to reduce the dimensionality of the parameters (18 of them) for ease of interpretation. The standardized principal component analysis (SPCA) method was applied to the 18 water quality parameters using the Statistical Package for Social Sciences (SPSS). The extracted principal components were subject to Varimax rotation in order to better distinguish the factor loadings on the parameters. Next, HCA was applied to the parameters as well as the sampling points. CG of parameters served as a confirmation for the results of the PCA. CG of sampling points were used for spatial delineation of water quality. HCA is the most widely used method for classifying a group of data into similar subgroups (Khound and Bhattacharyya 2016). In this paper, Ward’s method of linkage and squared Euclidean distance were employed. Finally, a hydrochemical classification of the water samples was attempted by generating Piper trilinear plots using Piperplot-QW.

## Results and discussion

### Physicochemical parameters of groundwater

Table S1 (in the Supplementary material) is the result of the water quality analysis obtained from the laboratory. In order to facilitate an in-depth interpretation of these results, the descriptive statistics of these parameters have been provided in Table 1. Table S1 shows that despite the geographical proximity of the sampling points, the water quality parameters exhibited wide variations from location to location. Fe^2+^, Mg^2+^, SO_4_^2−^, Cl^−^, total hardness (TH), Mn, Na^+^, SiO_2_, and alkalinity exhibited the highest levels of variation with coefficients of variation of 131.3, 92.8, 83.9, 76.7, 65.9, 64.3, 57.6, 57.2, 57.0, and 52.5 respectively. These wide variations may be due to the variabilities in the solubility of aquifer materials, recharge zones, and anthropogenic activities at the sampling points. Temperature and pH had the least degree of variability of 4.5 and 5.9, respectively. While the temperature of water is not of serious concern in terms of potable water quality, pH is a significant water quality parameter. pH indicates the strength of the water to react with acidic or alkaline materials in water (Islam et al. 2017) and is a controlling factor that determines the types of ion present in water. Hence, water with low (acidic) pH is more likely to dissolve aquifer materials. The average pH value was 6.76 with a skewness of − 0.5. This implies that a higher proportion of the water samples fell in the acid pH range. In fact, 71% of the water samples were slightly acidic. Generally, low water pH and high Fe^2+^ content are characteristics of the weathered basement complex rocks of southwestern Nigeria (Oke and Tijani 2012; Ufoegbune et al. 2009). Furthermore, a significant positive correlation exists between pH and Fe^2+^, thus indicating a possible influence of industrial activities on water quality. This result is supported by the findings of Romshoo et al. (2017), where he reported high variability of Fe^2+^ (mean pH 7.45) in the groundwater in Jammu Siwaliks, India due to iron release from industries.

**Table 1:** Descriptive analysis of water samples obtained from the study area (*n*^d^ = 63 from 21 different taps)

	pH	TDS (mg/l)	EC (μS/cm)	Temp (°C)	Alk (mg/l)	Cl^−^ (mg/l)	CO_3_^2−^ (mg/l)	HCO_3_^−^ (mg/l)	SO_4_^2−^ (mg/l)	
Mean	6.76	1135.86	1573.57	25.52	27.45	181.32	15.81	411.27	298.29	
Max	7.40	2126.00	3314.67	27.37	56.01	546.20	25.67	666.27	978.60	
Min	5.80	498.33	665.33	23.10	10.06	32.60	0.00	278.30	35.73	
SD	0.40	471.31	705.29	1.16	14.62	140.88	7.83	107.38	253.37	
Skewness	− 0.50	0.53	0.80	− 0.08	0.91	1.28	− 0.96	1.20	1.45	
Kurtosis	0.09	− 0.61	0.21	− 0.40	− 0.53	1.38	0.40	1.20	1.62	
Q1	6.53	725.00	1037.33	24.80	16.88	84.03	13.00	338.93	138.90	
Q3	7.01	1440.67	2011.00	26.23	29.67	231.37	22.33	457.87	321.57	
CV	5.9	40.3	44.3	4.5	52.5	76.7	48.9	25.8	83.9	
WHO	6.5–8.5	1000	1500	–	–	250	–	500	250	
Violations	5	11	10	N/A	N/A	4	Nil	3	9	
% violation	23.8	55.0	47.6	N/A	N/A	19.0	Nil	14.3	42.9	
	NO_3_^−^ (mg/l)	F^−^ (mg/l)	Na^+^ (mg/l)	K^+^ (mg/l)	Ca^2+^ (mg/l)	Mg^2+^ (mg/l)	Fe^2+^ (mg/l)	Mn (mg/l)	SiO_2_ (mg/l)	TH (mg/l)
Mean	3.43	1.23	193.46	5.80	78.48	89.91	0.52	0.03	3.67	566
Max	6.17	1.84	514.73	9.30	227.67	347.27	2.96	0.08	8.03	1740.3
Min	0.00	0.48	55.97	2.07	22.00	21.37	0.02	0.01	0.40	209.8
SD	1.99	0.42	112.88	2.00	43.52	84.46	0.69	0.02	2.12	377.4
Skewness	0.06	− 0.61	1.35	− 0.14	2.18	1.79	2.43	0.75	0.36	1.7
Kurtosis	− 1.29	− 0.70	2.43	− 0.82	6.28	3.18	7.33	− 0.47	− 0.74	3.4
Q1	2.00	1.03	124.23	4.53	55.67	37.63	0.04	0.02	2.20	299.7
Q3	5.73	1.55	213.97	7.70	95.00	107.77	0.75	0.04	5.37	857.6
CV	57.2	34.3	57.6	34.1	54.8	92.8	131.3	64.3	57.0	65.9
WHO	50	1.5	50	12	300	50	0.3	0.4	–	500
Violations	Nil	7	21	0	0	11	10	0	N/A	8
% violation	Nil	33.3	100	Nil	Nil	52.4	47.6	Nil	N/A	38.1

The degree of non-compliance of water quality parameters with WHO drinking water quality standards was computed as a percentage of the total number of times a parameter exceeded stipulated standards. It was found that Na^2+^, Mg^2+^, Fe^2+^, and EC exhibited the most violation of drinking water standards with percent violations of 100, 52.4, 47.6, and 47.6%, respectively. High levels of Na^+^ could be as a result of erosion of salt deposits and sodium-bearing rocks, groundwater pollution by sewage, irrigation, and precipitation leaching of soils high in sodium. Sodium concentration greater than 200 mg/L was observed in the samples collected from region R5, R1, R8, R2, R3, and R11 in their increasing order, respectively (Table S1). But when compared with Al-Qassim in central Saudi Arabia (El Alfy et al. 2018), Southern Tiruchirappalli District in India (Selvakumar et al. 2017), and Torbat-Zaveh Plain in Iran, the sodium content obtained in this study was lesser (Nematollahi et al. 2016). While Na^+^ is not of serious health concern with respect to drinking water, its presence in high concentration can lead to the deterioration of soil structure and reduced crop yield if the water is used for irrigation (Islam et al. 2017). The high levels of Fe^2+^ in the groundwater samples is probably due to the low water pH resulting in the corrosion of water delivery pipes and leaching of Fe^+^ from the weathered basement complex. Although Fe^2+^ is an essential element in humans and is of little health concern, its presence in water constitutes a nuisance (Nag and Das 2017). High levels of Fe^2+^ in drinking water can impart taste, stimulate bacterial growth, and cause stains on clothes, fittings, and utensils. Parameters, such as Mn, Ca^2+^, NO_3_^−^, and CO_3_^2−^, were always within the WHO guideline values for drinking water. Khound and Bhattacharyya (2016) observed that the solubility of Mn is high in low pH, but that does not seem to be the case in this study. They further observed that Fe^2+^ generally coexists with Mn in water, but the concentration of Fe^2+^ is always higher than Mn due to its crustal abundance. In line with this, it was observed that Fe^2+^ was higher than Mn in 86% of the samples analyzed. TDS and EC exhibited 55 and 47.6% violations of WHO guideline values (Table 1). TDS can be used as a firsthand assessment of the potability of water (Sharma et al. 2016). TDS level in water is dependent on the chemical nature of the water as well as the solubility of the aquifer materials through which the water is flowing. Aghazadeh et al. (2016) noted that high TDS and EC could be attributed to ion exchange, evaporation, sediment dissolution, and rainwater infiltration. Heavy use of agro-chemicals can also contribute to high levels of TDS in groundwater. Sharma et al. (2016) noted that high levels of groundwater TDS could result from the contribution of dissolved salts from the unsaturated zone. The high levels of TDS in the groundwater samples investigated should be a source of great concern. It has been established that high levels of TDS can lead to gastrointestinal irritations and laxative effects (Selvakumar et al. 2014).

The classification provided by Davis and De Wiest (1966) was further used to assess the potability of the water samples. Table 2 shows that only 47.62% of the water samples is fit for drinking, while the remaining 52.38% will be useful for irrigation. Regarding hardness, all the 21 samples were categorized as very hard with a TH of greater than 180 MgCaCO_3_/l. Hardness results from the presence of sulfate, chloride, and bicarbonates of calcium and magnesium. TH impairs the lather-forming ability of water thereby leading to wastage of water and detergent during laundry. Hard water is generally of little health concern, but can cause serious problems in industrial settings where it can lead to the breakdown of boilers, cooling towers, and other equipment as a result of scum formation (Ramya et al. 2015).

**Table 2:** Suitability of water samples for drinking

Water class		No. of sample in each class	% of sample in each class
TDS(mg/l)			
< 500	Desirable for drinking	1	4.76
500–1000	Permissible for drinking	9	42.86
1000–3000	Useful for irrigation	11	52.38
> 3000	Unfit for drinking	0	0
TH (mgCaCO_3_/l)			
< 60	Soft	0	0
60–120	Moderately hard	0	0
121–180	hard	0	0
> 180	Very hard	21	100

### Correlation of physicochemical parameters

Pearson’s correlation coefficients were computed for each pair of the parameters as shown in Table 3. A significant correlation was found to exist between Na^+^ and Cl^−^ (*r* = 0.84, *α* = 0.01). Significant and positive correlation was also obtained between TDS and cations, such as Mg^2+^ (*r* = 0.72, *α* = 0.01), Ca^2+^ (*r* = 0.49, *α* = 0.05), and Na^+^ (*r* = 0.57, *α* = 0.05), and anions, such as SO_4_^2−^ (*r* = 0.77, *α* = 0.01), HCO_3_^−^ (*r* = 0.65, *α* = 0.01), Cl^−^ (*r* = 0.58, *α* = 0.01), and SiO_2_ (*r* = −0.54, *α* = 0.01). This suggests that hardness-causing ions contributed a high proportion of the TDS. Further analysis showed that a significant positive correlation of 0.84 (*α* = 0.01) existed between TDS and TH. Nag and Das (2017) observed that impurities in limestone, such as SO_4_^2−^, Cl^−^, and SiO_2_, become exposed to the solvent action of water, as carbonates are dissolved so that they also pass into solution. This partly explains the high correlation between TDS and TH as well as Ca^2+^ and SO_4_^2−^ (*r* = 0.66, *α* = 0.01) and Cl^−^ (*r* = 0.46, *α* = 0.05). The high correlation coefficient between Na^+^ and Cl^−^ in groundwater samples is commonly reported in the literature and can be attributed to the dissolution of anhydrite, gypsum, and halite (Islam et al. 2017; Sreedhar et al. 2016; Li et al. 2013; Giridharan et al. 2009). The same also goes for the high correlation between Ca^2+^ and SO_4_^2−^. Potassium (K^+^) and NO_3_^−^ were not significantly correlated to any other ions apart from each other (*r* = 0.511, *α* = 0.05). This suggests that both ions enter groundwater from the same source and via the same route which is most likely fertilizer application for agricultural purposes. Fluoride and manganese were not correlated with any ion.

**Table 3:** Parameter correlation of hydrochemical data

	pH	TDS	EC	Temp.	Alk	F^−^	Fe^2+^	Mn	Ca^2+^	K^+^	Mg^2+^	Na^+^	SO_4_^2−^	SiO_2_	HCO_3_^−^	Cl^−^	NO_3_^−^	CO_3_^2−^
pH	1.00																	
TDS	0.43	1.00																
EC	0.38	0.91^**^	1.00															
Temp.	0.04	− 0.14	− 0.18	1.00														
Alk	0.46^*^	0.09	0.09	0.13	1.00													
F^−^	0.22	0.41	0.39	− 0.31	0.19	1.00												
Fe^2+^	0.50^*^	0.11	0.15	0.00	− 0.09	− 0.09	1.00											
Mn	0.54^*^	0.20	0.08	− 0.21	0.52^*^	0.05	0.07	1.00										
Ca^2+^	0.33	0.49 ^*^	0.64^**^	− 0.41	0.13	0.43	0.27	0.06	1.00									
K^+^	− 0.45^*^	0.19	0.34	− 0.01	− 0.42	− 0.14	− 0.16	− 0.35	0.24	1.00								
Mg^2+^	0.21	0.72 ^**^	0.57^**^	0.00	0.00	0.33	− 0.11	− 0.13	0.13	0.04	1.00							
Na^+^	0.17	0.47^*^	0.34	− 0.18	0.12	− 0.05	− 0.02	0.24	0.01	0.27	0.45^*^	1.00						
SO_4_^2−^	0.23	0.77^**^	0.79^**^	− 0.32	− 0.05	0.32	0.15	− 0.14	.660^**^	0.37	0.66^**^	0.51^*^	1.00					
SiO_2_	− 0.51^*^	− 0.54^*^	− 0.48^*^	0.31	0.05	− 0.11	− 0.34	− 0.29	− 0.21	− 0.08	− 0.37	− .472^*^	− 0.42	1.00				
HCO_3_^−^	0.20	0.65^**^	0.65^**^	− 0.38	− 0.28	0.02	0.28	− 0.09	0.40	0.36	0.43	0.39	0.65^**^	− 0.57^**^	1.00			
Cl^−^	0.34	0.58^**^	0.54^*^	− 0.28	0.09	0.06	0.18	0.20	0.38	0.34	0.46^*^	0.84^**^	0.76^**^	− 0.57^**^	0.49^*^	1.00		
NO_3_^−^	− 0.20	0.24	0.23	0.02	− 0.13	0.02	− 0.18	0.04	0.19	.511^*^	0.14	− 0.01	0.11	− 0.22	0.02	0.15	1.00	
CO_3_^2−^	− 0.02	− 0.28	− 0.15	0.17	− 0.18	− 0.36	0.33	− 0.14	− 0.43^*^	− 0.19	− 0.20	0.10	− 0.22	0.02	0.08	− 0.12	− 0.59^**^	1.00

^*^Correlation is significant at the 0.05 level (two-tailed)

^**^Correlation is significant at the 0.01 level (two-tailed)

### Principal component analysis of groundwater quality parameters

**Fig. 2:**
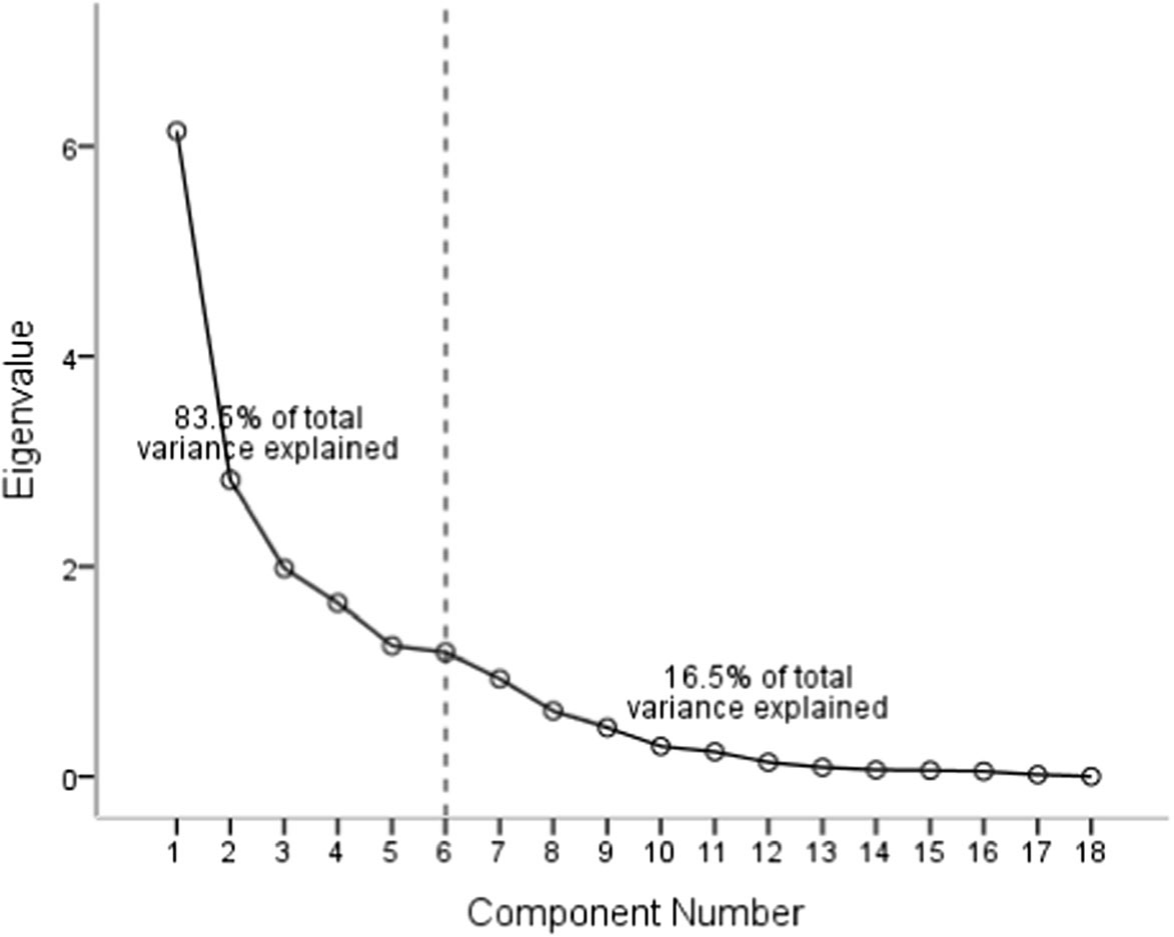
Scree plot of principal components

PCA aids the interpretation of complex multidimensional data matrices for a better understanding of water quality (Emenike et al. 2017a, b; Sreedhar et al. 2016). Six principal components whose Eigenvalues were greater than one were extracted from the 18 parameters. These six principal components accounting for 78% reduction in dimensionality explained 83.5% of the total variance (Fig. 2). The first principal component (PC1) explained 34.1% of the total variance and has a high positive loading on TDS (0.88), Mg^2+^ (0.87), SO_4_^2−^ (0.78), EC (0.77), F^−^ (0.54), and HCO_3_^−^ (0.51) as shown in Table 4. Obviously, PC1 represents the influence of mineral dissolution from geological formations on the hydrochemistry of groundwater. This implies that the quality of water in the study area is greatly dependent on the aquifer material. Oke and Tijani (2012) observed that weathering effect aided by abundant rainfall experienced in the area leads to continuous leaching of minerals into groundwater. Hence, it follows that mineral dissolution has more effect on the hydrochemistry of Abeokuta South than anthropogenic activities.

**Table 4:** Principal components of groundwater parameters

Parameter	Component					
	PC1 (34.1%)	PC 2 (15.68%)	PC 3 (11.01%)	PC 4 (9.16%)	PC 5 (6.92%)	PC 6 (6.58)
pH	0.32	0.12	0.67	− 0.13	0.57	− 0.03
TDS	0.88	0.30	0.10	0.15	0.18	0.04
EC	0.77	0.16	0.05	0.30	0.30	0.20
Temp	− 0.03	− 0.28	0.08	0.09	0.01	− 0.91
Alkalinity	0.06	0.00	0.85	0.03	− 0.11	− 0.04
F^−^	0.54	− 0.32	0.30	0.03	− 0.17	0.47
Fe^2+^	0.03	0.00	− 0.01	− 0.21	0.89	−0.01
Mn	− 0.14	0.47	0.75	− 0.02	0.15	0.10
Ca^2+^	0.48	− 0.12	0.05	0.33	0.38	0.57
K^+^	0.10	0.25	− 0.63	0.60	− 0.03	0.04
Mg^2+^	0.87	0.23	0.00	0.00	− 0.19	− 0.16
Na^+^	0.29	0.90	0.06	− 0.03	− 0.11	0.01
SO_4_^2−^	0.78	0.29	− 0.15	0.18	0.15	0.33
SiO_2_	− 0.25	− 0.54	− 0.17	− 0.22	− 0.48	− 0.17
HCO_3_^−^	0.51	0.39	− 0.36	− 0.01	0.41	0.27
Cl^−^	0.41	0.75	0.06	0.20	0.15	0.19
NO_3_^−^	0.09	0.13	− 0.15	0.88	− 0.03	− 0.13
CO_3_^2−^	− 0.31	0.11	− 0.20	− 0.71	0.30	− 0.22

**Fig. 3:**
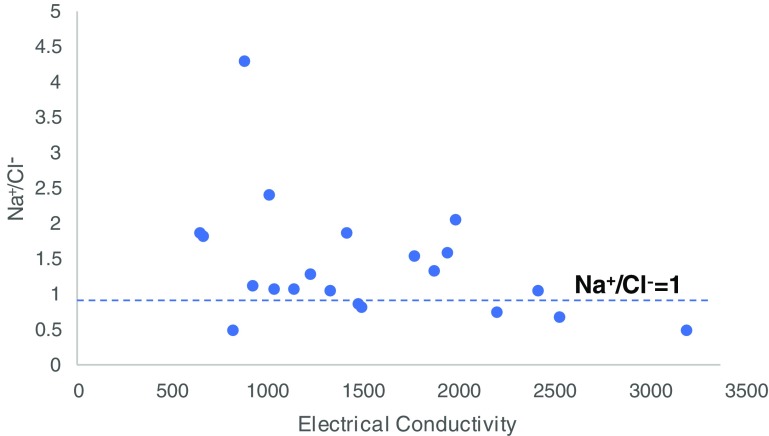
Na^+^/Cl^−^ ratio plot of groundwater samples

The second principal component (PC2) explained 15.7% of the total variance with high loadings on Na^+^ and Cl^−^. Both Na^+^ and Cl^−^ are widely distributed in nature as NaCl. These ions can enter water by weathering of rocks, agricultural chemicals, septic tank effluent, animal waste, municipal landfill leachate, seawater, basin brines, road deicers, and irrigation discharge (Bora and Goswami 2016; Panno et al. 2006). Etteieb et al. (2015) also observed that high levels of Na^+^ and Cl^−^ in water may be attributed to increase in industrial water pollution probably from uncontrolled discharge of industrial effluent. The relationship between Na^+^ and Cl^−^ is often used to identify the mechanism of acquiring salinity and to quantify atmospheric contribution (Tiwari and Singh 2014). The average Na^+^/Cl^−^ ratio of 1.4 (Fig. 3) suggests limited contribution from the atmospheric precipitation and reveals that the high levels these ions are most likely from weathering of rocks and anthropogenic sources. Only six of the samples have Na^+^/Cl^−^ < 1.0, indicating limited role of ion exchange from Ca^2+^ and Mg^2+^ in clays. With respect to the study area, Na^+^ and Cl^−^ might have been introduced into groundwater from municipal solid waste leachate, septic tank effluent, industrial effluent, and animal and agricultural waste. Hence, PC2 represents the influence of poor waste management on groundwater chemistry. PC3 has a high positive loading on alkalinity and pH and a high negative loading on K^+^. While it is not clear what this principal component represents, it seems to point to the influence of the natural environment (soil and air) on the chemistry of groundwater. PC4 has a high loading on K^+^ and NO_3_^−^ with an explained variance of 9.16%. This component obviously represents the impact of agricultural practices on groundwater quality. Common sources of NO_3_^−^ are fertilizers, domestic waste, sewage sludge used for agricultural purposes, and organic matter (Barzegar et al. 2017; Sharma et al. 2016). PC5 has a high positive loading on Fe^2+^. The poor correlation between Fe^2+^ and Mn (0.07) implies that the two metals are not from the same source and therefore suggests that a significant proportion of Fe^+^ did not emanate from the aquifer materials. It was earlier inferred that the high concentrations of Fe^2+^ in the water samples could have been as a result of the corrosion of conveyance pipes and pump materials by water of low pH. Hence, this component most likely represents the influence of pumping and conveyance on water quality. PC6 has a high negative loading on temperature (*r* = − 0.91) and high positive loadings of 0.57 and 0.47 on Ca^2+^and F^−^, respectively. Hence, PC6 indicates the contributions of the dissolution of paleosols and quartzite near the groundwater table (Chuah et al. 2016; Emenike et al. 2018; Xiao et al. 2015).

### Geospatial variation of water quality

**Fig. 4:**
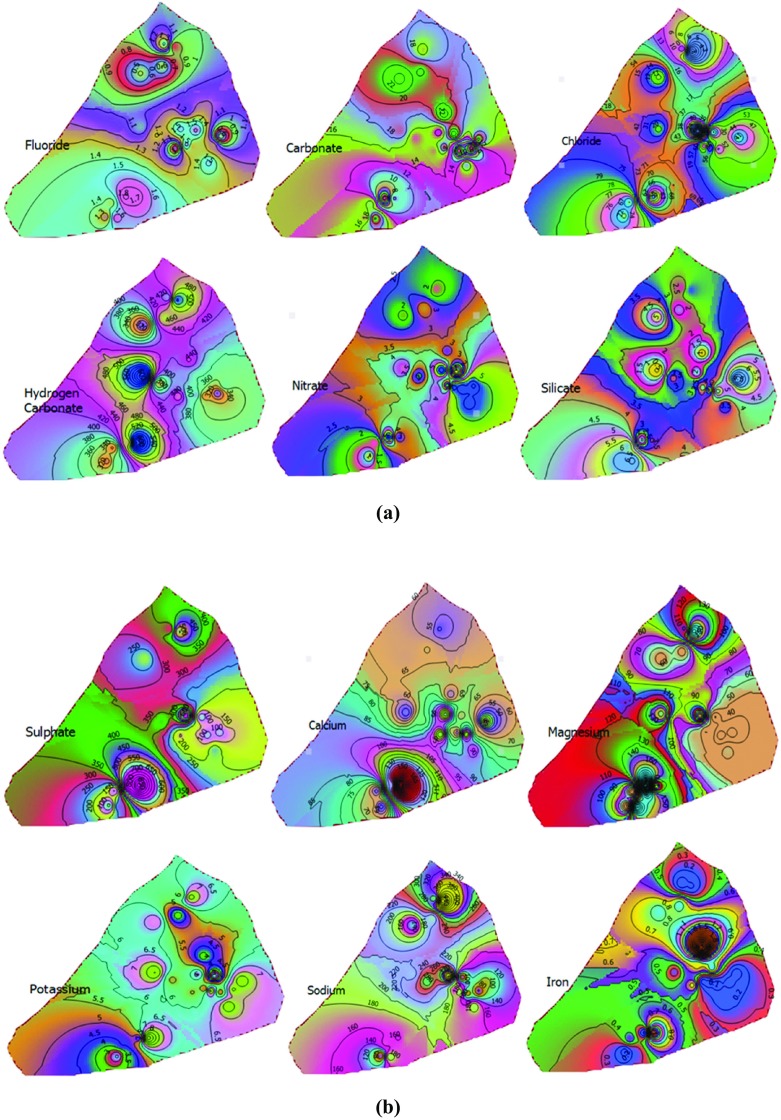
**a** Geospatial distribution of SiO_2_, CO_3_^2−^, HCO_3_^−^, Cl^−^, NO_3_^−^, and F^−^. **b** Geospatial distribution of SO_4_^2−^, Ca^2+^, Na^+^, Mg^2+^, K^+^, and Fe^2+^

**Fig. 5:**
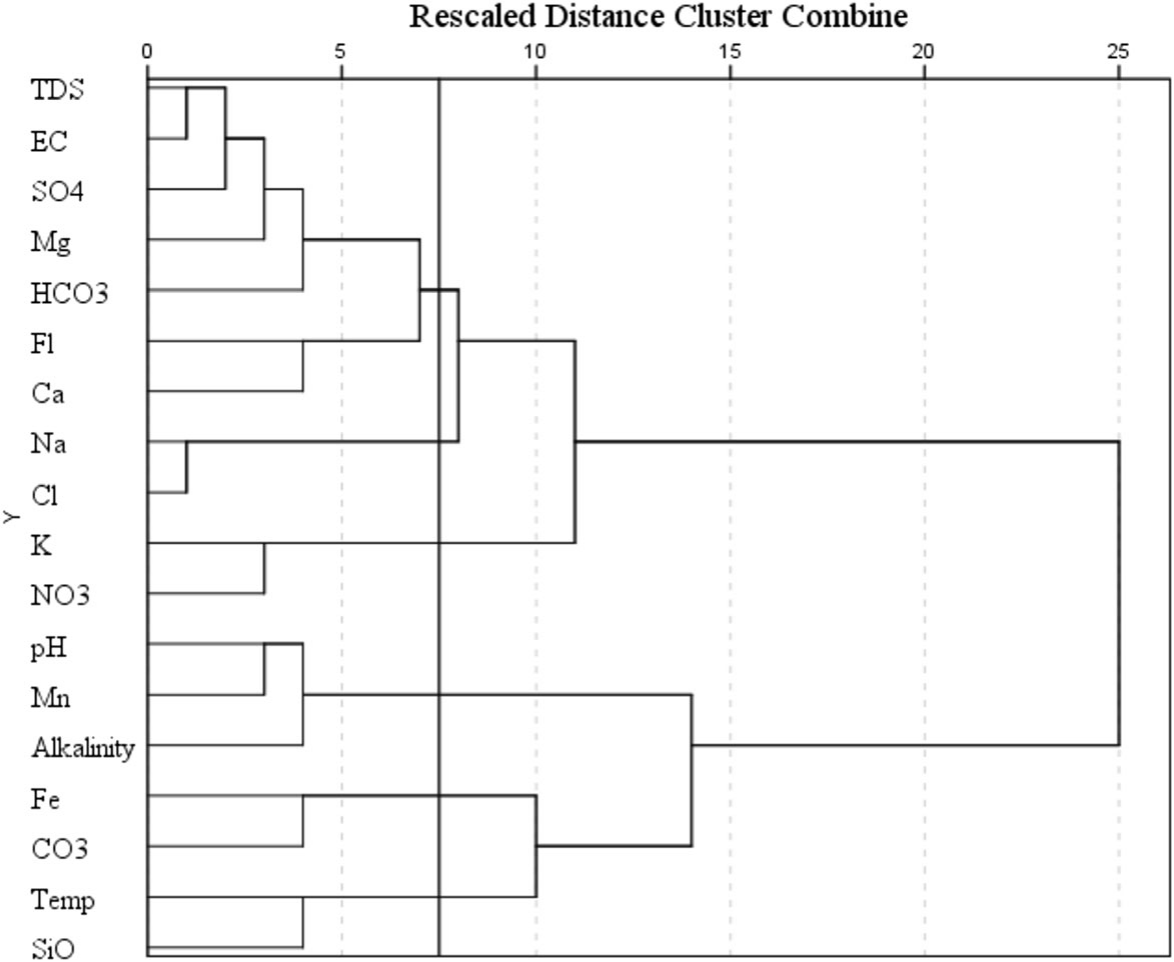
Hierarchical cluster groupings based on water quality parameters

Figure 4 shows that groundwater in Abeokuta South exhibited a high degree of spatial variability. Most of the ions, such as Cl^−^, Mg^2+^, SiO_2_, HCO_3_^−^, F^−^, Ca^2+^, SO_4_^−^, and K^+^, recorded the highest values in the southern part of the study area. The highest concentration of Na^+^ occurred in the northern part while the highest concentrations of Fe^2+^, CO_3_^−^, and HCO_3_^−^ occurred closer to the city center. In order to enhance the interpretation of these geospatial maps, cluster analysis was applied to the hydrochemical parameters obtained from all the sampling points (Fig. 5). The analysis yielded four hydrochemical clusters of the study area with characteristics shown in Table 5. The average concentrations of water quality parameters were compared with WHO guideline values for drinking water. Clusters 3 and 4 exhibited the worst violations of water quality standards with percent violation of 57.1 and 64.3%, respectively. Both of these clusters had average TDS concentration greater than 1000 mg/l, which renders them only useful for irrigation as per the classification of Davis and De Wiest (1966). Cluster 2 was found to possess the best hydrochemical quality followed by cluster 1 with percent violations of 13.3 and 26.7%, respectively. Despite the relatively good groundwater quality of cluster 2, it was found that all but one of the groundwater samples from this cluster had pH values less than 6.5. The decreasing order of water quality was cluster 2 > cluster 1 > cluster 3 > cluster 4. Based on the hierarchical CG, a composite geospatial map of the study area was produced. Some tap water gets contaminated at the source and also after treatment unknowing to the consumers due to negligence to identify possible contamination points before being supplied publicly or privately (Tenebe et al. 2017). These points of contamination are mainly from indiscriminate discharge from anthropogenic, commercial, or industrial activities (Olusheyi 2017; Tenebe et al. 2018). With this in mind, the variability of water quality within the study area was largely associated with the distinct individual and cooperate activities engaged by inhabitants within the as reported in the literature (Table 6).

**Table 5:** Hydrochemical characteristics of cluster groupings

Parameter	Cluster 1	Cluster 2	Cluster 3	Cluster 4	WHO limit
pH	6.8	6.3	7.0	7.1	6.5–8.0
TDS (mg/l)	875.3	834.2	1286.9	1833.3	1000
EC (μS/cm)	1154.4	1235.6	1638.5	2678.8	1500
Temp (°C)	25.6	25.8	25.4	25.3	–
Alka (mg/l)	34.3	18.2	22.8	38.0	–
F^−^ (mg/l)	1.5	1.1	0.8	1.7	1.5
Fe (mg/l)	0.3	0.2	1.0	0.6	0.3
Mn (mg/l)	0.03	0.02	0.0	0.0	0.4
Ca^2+^ (mg/l)	60.0	62.3	66.8	132.2	300
K^+^ (mg/l)	3.6	7.3	5.8	6.5	12
Mg^2+^ (mg/l)	69.5	46.3	108.6	174.6	50
Na^+^ (mg/l)	128.2	159.8	269.9	257.2	50
SO_4_^2−^ (mg/l)	128.9	202.4	316.5	687.1	250
SiO_2_ (mg/l)	5.4	4.5	1.7	2.2	–
HCO_3_^−^ (mg/l)	352.3	337.5	516.6	485.8	500
Cl^−^ (mg/l)	56.6	133.6	250.3	349.1	250
NO_3_^−^ (mg/l)	1.2	4.5	3.3	4.1	50
CO_3_^2−^ (mg/l)	20.1	15.4	19.8	10.1	–
TH (mg/l)	435.9	345.9	615.9	1048.6	500
% violation	26.7	13.3	57.1	64.3	–

**Table 6:** Causes and water quality status in Abeokuta environment

Location	Pollution sources	Pollutants identified	References
Kotopo and Ajebo	Mechanic site	Fe, nitrates (high)	Olusheyi (2017)
Lafenwa	Abattoir, sawmill, Locust beans processing factories	Chloride, nitrate, phosphate	Ojekunle et al. (2016)
Alabata Road	Poultry and dumpsite waste	pH and EC (high)	Taiwo et al. (2013)

**Fig. 6:**
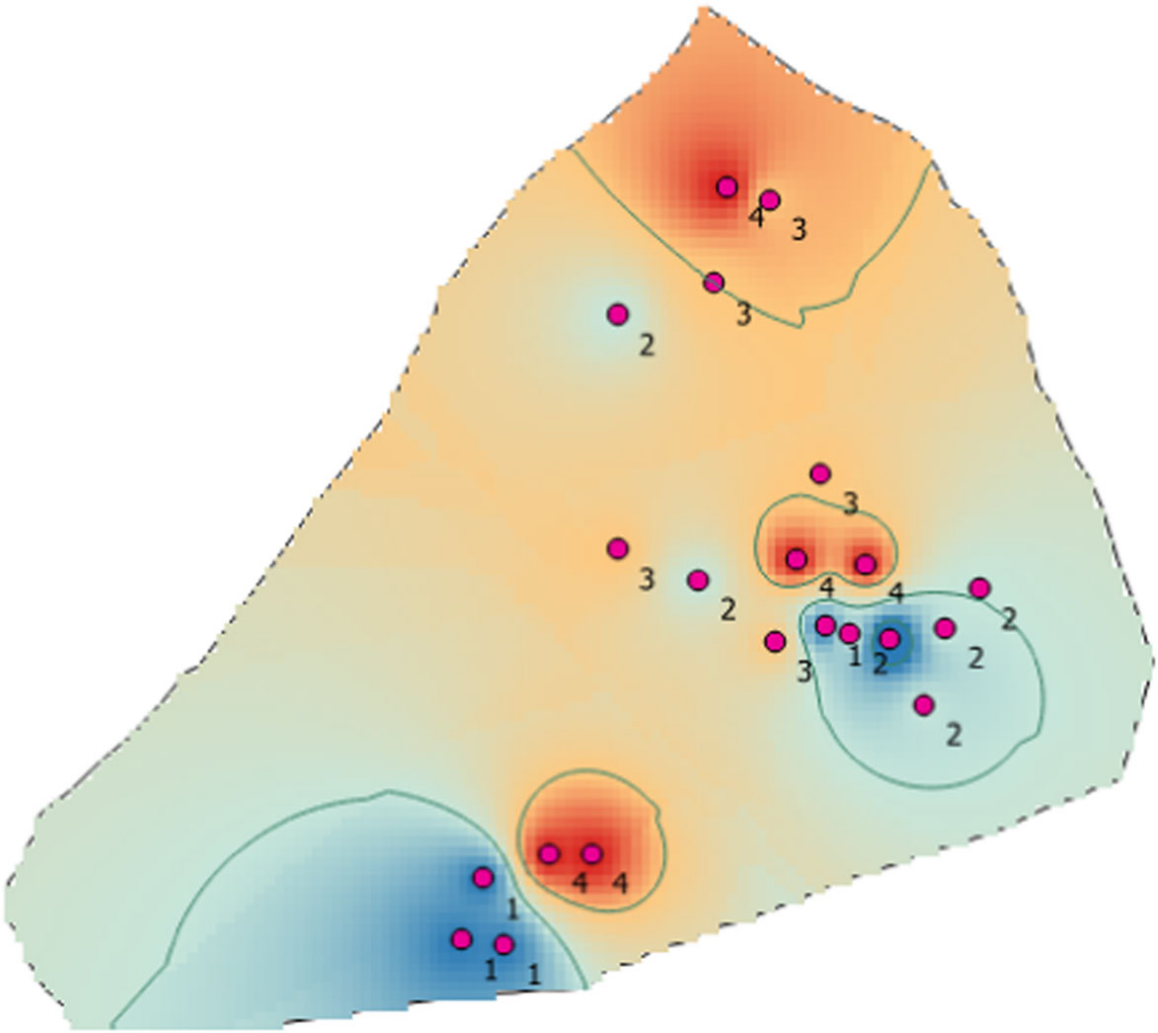
Composite geospatial map of water quality in Abeokuta South

Figure 6 shows that the best groundwater quality exists in the eastern and southwestern areas of the study location, while worst groundwater quality exists at the city center. This again points to the significant influence of anthropogenic activities on groundwater quality. Urban areas are prone to elevated nutrient levels as a result of industrial, residential, and agricultural practices (Sarukkalige 2011). Industries contributing to water pollution in Abeokuta South includes sawmills, breweries, abattoirs, agricultural practices, automobile shops, and numerous illegal solid waste dumps in the municipality.

### Water quality assessment

**Fig. 7:**
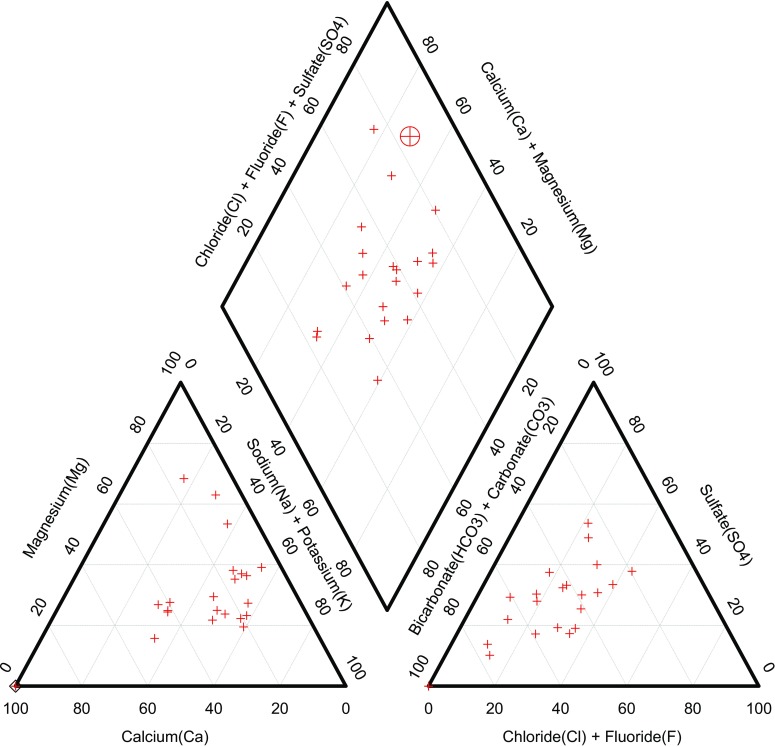
Piper diagram showing the hydrological facies of the groundwater samples

The Piper-trilinear plot (Piper 1944) displays the classification of water samples from different lithological environment. It also represents the chemical character of the water samples using the dominant cation and anion to tell the differences and similarities of the groundwater samples. In this study, chemical data was plotted on a Piper diagram (Fig. 7), and the results revealed that 38% of the samples could be classified as Na-K-HCO_3_ type, 29% of the samples as Na-K-Cl-SO_4_ type, 24% of the samples as Ca-Mg-HCO_3_ type, and 9% as Ca-Mg-Cl-SO_4_ type. The results also suggest the dominance of Na which could be as a result of weathering of rocks (Xiao et al. 2015). Furthermore, it could be said that multiple processes contribute to the composition of the hydrochemical facies as this can be from the mixed groundwater types. The Piper plot (Fig. 7) also revealed Na^+^ and K^+^ dominance in the cation composition, while HCO_3_^2−^ and SO_4_^2−^ dominate the anion composition in the groundwater samples.

## Conclusion

This study reported the levels of groundwater quality parameters used for agricultural, domestic, and drinking purposes in Abeokuta, Nigeria. The results revealed that the water quality parameters showed wide spatial variations with pH and temperature having the least variability of 5.9 and 4.5, respectively. Most of the water samples (71%) fell within the slightly acidic range indicating dissolution of complex basement rocks. Violations of water standards were in order of Na > Mg > Fe > EC, which suggests interaction with sodium-bearing rocks. The groundwater groupings can be ranked as Ca-Mg-Cl-SO_4_ > Ca-Mg-HCO_3_ > Na-K-Cl-SO_4_ > Na-K-HCO_3_ indicating mixed type. Mineral dissolution from soil and aquifer as well as anthropogenic activities, such as agriculture and waste management, were the major sources of hydrochemical variation in the study area. The very high concentration of TDS in the water samples were identified as a serious source of health concern. The findings of this research will be beneficial to water management authorities to understand the hydrochemistry of the groundwater potentials in the region for efficient and sustainable management.

## Electronic supplementary material

ESM 1 (DOCX 35 kb)
