# Supplementary material for: Assessment of geospatial and hydrochemical interactions of groundwater quality, southwestern Nigeria
Source: Environ Monit Assess. 2018 Jun 28;190(7):440. doi: 10.1007/s10661-018-6799-8 (PMC6022516; doi:10.1007/s10661-018-6799-8)
Supplement: Supplementary file 1 — (DOCX 35 kb) [file 10661_2018_6799_MOESM1_ESM.docx]

**Supplementary Material**

Table S1: physico–chemical parameters of individual tap water obtained from the study area (n^d^ = 63 from 21 different taps)

| **Sample** | **Stat** | **pH** | **TDS (mg/l)** | **EC (µS/cm)** | **Temp (^o^C)** | **Alka (mg/l)** | **Cl^–^ (mg/l)** | **CO_3_^2–^ (mg/l)** | **HCO_3_^–^ (mg/l)** | **SO_4_^2–^ (mg/l)** | **NO_3_^–^ (mg/l)** | **F^–^ (mg/l)** | **Na^+^ (mg/l)** | **K^+^ (mg/l)** | **Ca^2+^ (mg/l)** | **Mg^2+^ (mg/l)** | **Fe^2+^ (mg/l)** | **Mn (mg/l)** | **SiO_2_ (mg/l)** |
| --- | --- | --- | --- | --- | --- | --- | --- | --- | --- | --- | --- | --- | --- | --- | --- | --- | --- | --- | --- |
| **R1** | **Mean** | 6.53 | **1440.67** | **2011.00** | 24.43 | 13.01 | 134.20 | 19.33 | 659.27 | 319.57 | 4.40 | 1.03 | 213.97 | 8.03 | 46.67 | **165.10** | 0.46 | 0.02 | 0.40 |
|  | **SD** | 0.02 | 735.60 | 1382.18 | 0.06 | 0.01 | 12.38 | 4.93 | 152.81 | 27.00 | 1.11 | 0.00 | 28.53 | 0.81 | 5.51 | 7.88 | 0.06 | 0.00 | 0.44 |
| **R2** | **Mean** | **6.22** | 949.00 | **1262.00** | 23.10 | 26.55 | 226.27 | 14.33 | 338.93 | 400.10 | 2.30 | 1.31 | 295.03 | 5.40 | 73.23 | **76.00** | 0.24 | 0.05 | 3.67 |
|  | **SD** | 0.01 | 393.39 | 858.41 | 0.01 | 0.06 | 13.05 | 3.06 | 33.38 | 21.67 | 2.21 | 0.02 | 7.89 | 0.17 | 11.95 | 10.11 | 0.03 | 0.00 | 0.55 |
| **R3** | **Mean** | 7.21 | **1453.33** | **2274.00** | 24.97 | 56.01 | 546.20 | 13.00 | 405.40 | 659.87 | 5.87 | **1.55** | 410.23 | 6.57 | 118.33 | **158.20** | 0.72 | 0.06 | 1.47 |
|  | **SD** | 0.06 | 702.52 | 1401.89 | 0.06 | 0.02 | 22.23 | 2.65 | 12.91 | 73.38 | 0.86 | 0.02 | 47.13 | 0.75 | 8.50 | 17.72 | 0.01 | 0.00 | 0.15 |
| **R4** | **Mean** | 6.91 | 679.33 | 665.33 | 25.20 | 23.93 | 33.50 | 25.67 | 379.03 | 62.27 | 0.00 | **1.65** | 63.13 | 2.07 | 67.23 | 28.50 | 0.88 | 0.04 | 3.37 |
|  | **SD** | 0.01 | 47.16 | 92.64 | 1.59 | 2.37 | 8.81 | 4.16 | 24.31 | 10.08 | 0.00 | 0.10 | 7.90 | 0.15 | 2.36 | 0.82 | 0.09 | 0.00 | 0.59 |
| **R5** | **Mean** | 7.40 | **1290.67** | **1528.33** | 26.23 | 17.43 | 231.37 | 25.33 | 442.60 | 240.63 | 2.80 | 1.10 | 201.77 | 3.80 | 57.33 | **87.97** | 2.96 | 0.04 | 0.73 |
|  | **SD** | 0.01 | 540.63 | 743.09 | 0.32 | 1.82 | 17.31 | 7.37 | 51.84 | 13.38 | 0.61 | 0.11 | 8.17 | 0.30 | 4.04 | 4.25 | 0.07 | 0.00 | 0.51 |
| **R6** | **Mean** | 6.98 | 901.33 | **1463.67** | 24.95 | 18.30 | 84.03 | 21.00 | 379.50 | 175.03 | 2.00 | **1.59** | 157.33 | 5.10 | 67.00 | 39.07 | 0.03 | 0.04 | 4.50 |
|  | **SD** | 0.01 | 228.46 | 554.56 | 0.13 | 7.78 | 6.73 | 3.00 | 12.00 | 25.38 | 0.79 | 0.31 | 10.42 | 0.82 | 4.58 | 3.46 | 0.02 | 0.01 | 1.11 |
| **R7** | **Mean** | **6.43** | 932.33 | **1372.67** | 24.80 | 10.06 | 185.47 | 13.00 | 400.23 | 138.90 | 6.13 | 1.29 | 197.50 | 7.80 | 55.67 | 42.60 | 0.02 | 0.04 | 2.20 |
|  | **SD** | 0.02 | 154.62 | 351.44 | 1.76 | 0.26 | 11.55 | 2.00 | 12.04 | 10.87 | 0.83 | 0.21 | 42.41 | 1.87 | 7.02 | 4.84 | 0.01 | 0.01 | 0.30 |
| **R8** | **Mean** | 7.21 | **1234.00** | **1938.33** | 26.13 | 46.62 | 161.43 | 14.67 | 457.87 | 144.30 | 4.17 | 0.66 | 215.13 | 5.47 | 115.33 | **51.50** | 0.75 | 0.07 | 2.63 |
|  | **SD** | 0.01 | 117.36 | 250.59 | 0.15 | 1.19 | 21.07 | 2.52 | 37.65 | 13.36 | 0.71 | 0.09 | 8.27 | 0.35 | 10.50 | 5.57 | 0.07 | 0.02 | 1.33 |
| **R9** | **Mean** | 7.01 | **1098.33** | **1539.33** | 24.80 | 24.07 | 247.03 | 22.33 | 478.83 | 321.57 | 3.30 | 0.48 | 204.03 | 3.77 | 65.33 | 42.63 | 0.85 | 0.04 | 1.77 |
|  | **SD** | 0.01 | 335.32 | 675.36 | 0.66 | 0.05 | 7.46 | 9.29 | 70.72 | 16.46 | 0.44 | 0.07 | 9.60 | 0.50 | 4.73 | 1.75 | 0.07 | 0.01 | 0.78 |
| **R10** | **Mean** | 7.32 | **1897.67** | **2506.33** | 26.60 | 49.33 | 267.97 | 15.00 | 403.33 | 315.00 | 2.30 | 1.45 | 286.70 | 5.57 | 58.00 | **107.77** | 0.02 | 0.08 | 2.23 |
|  | **SD** | 0.02 | 1055.88 | 467.92 | 0.56 | 3.51 | 42.37 | 7.81 | 13.74 | 29.87 | 1.35 | 0.40 | 32.43 | 0.32 | 6.56 | 16.05 | 0.01 | 0.01 | 0.45 |
| **R11** | **Mean** | 6.60 | **1371.00** | **1175.67** | 25.52 | 13.02 | 477.70 | 17.33 | 544.47 | 556.20 | 1.63 | 0.81 | 514.73 | 7.70 | 49.33 | **195.67** | 0.08 | 0.02 | 3.00 |
|  | **SD** | 0.02 | 1162.25 | 224.00 | 0.33 | 1.06 | 38.56 | 6.66 | 24.56 | 90.82 | 1.76 | 0.14 | 53.49 | 0.18 | 7.02 | 7.98 | 0.02 | 0.00 | 0.62 |
| **R12** | **Mean** | 6.72 | 588.67 | **1069.00** | 27.33 | 16.69 | 114.67 | 24.67 | 278.30 | 206.57 | 1.87 | 0.48 | 124.23 | 6.85 | 62.67 | 44.20 | 0.84 | 0.01 | 5.37 |
|  | **SD** | 0.02 | 241.38 | 652.54 | 0.15 | 0.27 | 5.33 | 9.71 | 34.17 | 10.57 | 1.76 | 0.09 | 9.75 | 0.97 | 4.51 | 1.67 | 0.04 | 0.00 | 0.31 |
| **R13** | **Mean** | 6.86 | 725.00 | 906.67 | 27.37 | 54.41 | 38.17 | 16.67 | 315.60 | 92.93 | 1.50 | 1.35 | 164.63 | 4.53 | 52.33 | 29.13 | 0.58 | 0.02 | 5.90 |
|  | **SD** | 0.05 | 210.97 | 182.78 | 0.23 | 1.04 | 3.26 | 2.08 | 45.83 | 17.21 | 0.79 | 0.14 | 9.56 | 0.67 | 3.21 | 5.80 | 0.04 | 0.00 | 0.53 |
| **R14** | **Mean** | 6.71 | 498.33 | 682.67 | 24.47 | 48.60 | 32.60 | 14.67 | 310.90 | 35.73 | 0.43 | 1.23 | 60.03 | 2.37 | 66.00 | 24.03 | 0.04 | 0.03 | 6.43 |
|  | **SD** | 0.01 | 79.41 | 136.06 | 0.06 | 0.36 | 3.90 | 3.06 | 32.37 | 10.45 | 0.21 | 0.61 | 15.02 | 0.35 | 5.57 | 1.52 | 0.00 | 0.00 | 0.31 |
| **R15** | **Mean** | 6.61 | **1572.67** | **2053.67** | 26.17 | 26.30 | 94.87 | 22.33 | 376.27 | 278.63 | 2.27 | **1.66** | 195.90 | 4.07 | 47.33 | **226.97** | 0.20 | 0.02 | 6.70 |
|  | **SD** | 0.01 | 933.09 | 953.03 | 0.12 | 0.61 | 9.51 | 5.51 | 140.56 | 36.77 | 0.80 | 0.06 | 13.80 | 0.21 | 4.04 | 18.33 | 0.01 | 0.00 | 0.53 |
| **R16** | **Mean** | 7.13 | **2126.00** | **2620.00** | 25.97 | 29.67 | 231.13 | 0.00 | 468.00 | 794.83 | 5.73 | **1.84** | 158.37 | 4.60 | 124.67 | **347.27** | 0.05 | 0.02 | 1.33 |
|  | **SD** | 0.03 | 1256.10 | 1239.27 | 0.06 | 3.06 | 23.88 | 0.00 | 48.15 | 151.38 | 1.50 | 0.73 | 9.11 | 0.26 | 5.51 | 19.34 | 0.01 | 0.00 | 0.31 |
| **R17** | **Mean** | 6.90 | **1856.00** | **3314.67** | 23.77 | 16.88 | 351.13 | 12.33 | 666.27 | 978.60 | 2.57 | **1.76** | 173.33 | 9.30 | 227.67 | 85.27 | 1.51 | 0.01 | 3.57 |
|  | **SD** | 0.02 | 1425.28 | 2938.56 | 0.25 | 1.64 | 26.75 | 4.16 | 147.45 | 245.69 | 2.46 | 0.03 | 15.64 | 0.53 | 16.65 | 7.36 | 0.05 | 0.00 | 0.31 |
| **R18** | **Mean** | **5.80** | 726.33 | **1037.33** | 27.37 | 15.19 | 37.97 | 22.67 | 373.77 | 159.63 | 4.83 | 0.49 | 91.27 | 7.97 | 22.00 | 37.63 | 0.03 | 0.01 | 8.03 |
|  | **SD** | 0.03 | 139.47 | 329.22 | 0.55 | 3.37 | 6.57 | 4.04 | 36.23 | 23.33 | 4.24 | 0.36 | 7.09 | 0.59 | 2.65 | 7.64 | 0.01 | 0.00 | 0.68 |
| **R19** | **Mean** | **6.41** | 578.67 | 840.67 | 26.37 | 17.13 | 111.00 | 0.00 | 278.37 | 78.00 | 6.17 | 1.25 | 55.97 | 7.60 | 65.00 | 23.07 | 0.03 | 0.01 | 4.80 |
|  | **SD** | 0.02 | 154.11 | 284.91 | 0.06 | 0.15 | 11.85 | 0.00 | 34.88 | 4.35 | 1.47 | 0.01 | 8.43 | 0.46 | 3.61 | 3.15 | 0.00 | 0.00 | 0.66 |
| **R20** | **Mean** | **6.22** | **1230.33** | 1831.67 | 25.61 | 23.48 | 126.03 | 17.67 | 355.60 | 231.40 | 5.77 | **1.54** | 194.67 | 7.97 | 95.00 | 54.23 | 0.11 | 0.01 | 3.13 |
|  | **SD** | 0.02 | 180.45 | 293.27 | 0.10 | 0.02 | 11.32 | 2.89 | 17.91 | 18.60 | 1.40 | 0.01 | 23.85 | 0.40 | 6.00 | 3.67 | 0.00 | 0.00 | 0.35 |
| **R21** | **Mean** | 6.80 | .. | 952.00 | 24.80 | 29.67 | 75.03 | 0.00 | 324.10 | 74.37 | 6.03 | 1.33 | 84.73 | 5.20 | 112.00 | 21.37 | 0.54 | 0.07 | 5.87 |
|  | **SD** | 0.02 | 242.12 | 390.20 | 0.10 | 1.15 | 10.60 | 0.00 | 31.30 | 8.22 | 1.40 | 0.01 | 11.29 | 0.56 | 4.58 | 1.01 | 0.01 | 0.00 | 0.57 |
